# Local Governments’ Disaster Emergency Communication and Information Collection for Nutrition Assistance

**DOI:** 10.3390/ijerph16234617

**Published:** 2019-11-21

**Authors:** Noriko Sudo, Miyu Urakawa, Nobuyo Tsuboyama-Kasaoka, Kanami Yamada, Yoshiyuki Shimoura, Nobuo Yoshiike

**Affiliations:** 1 Natural Science Division, Faculty of Core Research, Ochanomizu University, Tokyo 112-8610, Japan; 2 Nutrition and Food Science Course, Faculty of Human Life and Environmental Sciences, Ochanomizu University, Tokyo 112-8610, Japan; miyu.urakawa0208@gmail.com (M.U.); k.n.min3@gmail.com (K.Y.); 3 Section of Global Disaster Nutrition, International Center for Nutrition and Information, National Institute of Health and Nutrition, National Institutes of Biomedical Innovation, Health and Nutrition, Tokyo 162-8636, Japan; ntsubo@nibiohn.go.jp; 4 The Japan Dietetic Association, Tokyo 105-0004, Japan; shimoura@dietitian.or.jp; 5 Department of Nutrition, Aomori University of Health and Welfare, Aomori City 030-8505, Japan; nobuoyos@muc.biglobe.ne.jp

**Keywords:** preparedness, information communication, disaster, local disaster management plan, disaster management radio communication, specified feeding facility

## Abstract

We examined local governments’ disaster emergency communication and information collection and distribution systems. Postal surveys were conducted for all prefectures, cities with public health centers, and specified districts in Tokyo Metropolis in 2005 and 2013. Municipalities were included in the 2013 survey only. The response rate for the 2013 survey was 71.2% (n = 1272). Thirty-six prefectures, 41 cities with health centers, and 16 specified districts in Tokyo Metropolis answered both surveys. A majority of respondents (88.8% in 2005 and 92.1% in 2013) of respondents reported that disaster management radio broadcasting was written into their local disaster management plans, guidelines, or manuals as the main communication tool. The proportion of respondents using computer networks (41.6–60.7%) and cell phones (email) (40.4–62.9%) had significantly increased between the surveys. It was also found that municipalities that had been previously affected by disasters (39.6%) were more likely to have systems to collect information from shelters and affected communities than those without any experience (24.3%), and prefectures that had been previously affected by disasters were more likely to have food supply damage reporting systems (36.4%) than those without such experience (3.3%).

## 1. Introduction

Over the past few decades, Japan has experienced several large-scale disaster events. When a disaster occurs, the responsible front-line organizations are local municipalities. However, because municipalities have minimal autonomy, they usually have limited manpower and economic resources. Therefore, in most cases, as dealing with the disaster situation and the aftermath is beyond their capacity, they need to give damage reports to prefectures when seeking additional support, which requires them to thoroughly assess the level of damage from information collected from affected residents, shelters, and communities.

When municipalities need external support, prefectures request assistance from central government ministries, agencies such as the self-defense forces, and other rescue organizations such as the Japanese Red Cross Society. Based on the specific offers of support, a prefecture will then allocate assistance depending on the needs of each municipality. The Social Welfare Councils at the municipality level then coordinate the general volunteers.

Under Japan’s Health Promotion Law, the public health centers in some prefectures, cities, and specified districts in Tokyo Metropolis are responsible for the supervision and provision of technical assistance to “specified food supply facilities” that are able to provide a large number of meals (100 meals at once or 250 meals per day). Although these food supply facilities have their own management, public health centers are required to ensure that these facilities and the food they serve are safe. This is especially important in hospitals and nursing homes, as even during disasters, hospital patients and other vulnerable people must eat three times a day to maintain their strength, which is why these food supply facilities must report any damage to the public health centers that administer them.

Communicating information from municipalities to residents is crucial in a disaster. A survey of evacuees in Miyagi Prefecture in the 2011 Great East Japan Earthquake found that a majority of respondents evacuated when they “heard the tsunami warnings” [1]. When a landslide occurred in Hiroshima in 2014, because the administration did not issue timely evacuation orders, 74 residents lost their lives. After evacuation shelters are opened, municipalities are responsible for providing people with information about, for example, missing family members and the locations of services such as food and water tank trucks [2]. As this information is critical in affected areas, shelters serve as information centers not only for the evacuees but also for those who have stayed in their homes [3].

To ensure effective disaster information flow, local governments and municipalities generally have several types of information communication systems. Disaster prevention administrative radio has both broadcast systems, which can be used with outside amplification equipment to directly convey disaster information such as evacuation orders to residents, and portable systems, which are used to share information across the various administrative bodies and related organizations. Disaster prevention administrative radios have been the main emergency communication tools for a longtime and they were also used in the Great East Japan Earthquake [4].

In the 2016 Kumamoto Earthquake, several communication avenues were used [5]. As reported by the National Institute of Information and Communications Technology, affected residents sent information about the disaster using social networking services (SNSs) such as Twitter, which was then used by emergency management centers to send resources and support [6]. From this experience, SNS has been seen as a promising system for the collection and sharing of emergency information. On the other hand, a young man who spread a false rumor about an escaped lion via Twitter was arrested for posting disinformation on the Internet during a disaster [4].

This study examined the communication tools used by various government bodies in a nationwide questionnaire survey in 2005 [7]. However, because of significant advances in information technology, a further survey was conducted in 2013, which allowed for a comparison of the disaster communication processes in Japan before and after the Great East Japan Earthquake.

## 2. Materials and Methods

### 2.1. Data Collection

The surveys administered in 2005 and 2013 were analyzed and compared.

#### 2.1.1. 2005 Survey

The census method was used and questionnaires were sent to the heads of health departments in 47 prefectures, 57 cities with public health centers (14 designated cities, 35 core cities, and eight major cities with health centers), and 23 specified districts in Tokyo Metropolis in November 2005. The response rate was 88.2% and completed questionnaires were returned from 41 prefectures, 50 cities with health centers (13 designated cities, 31 core cities, and six major cities with health centers), and 21 specified districts in Tokyo Metropolis.

#### 2.1.2. 2013 Survey

Surveys were again sent in September 2013 with a response deadline in mid-November 2013 to the health departments in 47 prefectures, 70 cities with health centers (20 designated cities, 42 core cities, eight major cities with health centers), and 23 specified districts in Tokyo Metropolis, and the 47 prefectures were also asked to distribute the surveys to their respective municipalities (total 1647). The census method was used. The authorities that did not initially respond on time were asked again and the response deadline was extended to February 2014. The response rate for the 2013 survey was finally 71.2% (42 prefectures, 59 cities with health centers (17 designated cities, 35 core cities, seven major cities with health centers), 18 specified districts in Tokyo Metropolis, and 1153 municipalities).

### 2.2. Survey Items

#### 2.2.1. Analyzed Items in the 2013 Survey

The survey had 13 sections. However, this study only focused on the two sections that asked questions associated with the gathering and provision of disaster information, as the other sections have been reported elsewhere [8,9,10,11,12].

The first section gathered information about responding authorities, in which they were asked if they had experienced and suffered damage from specific great earthquakes (Great Hanshin Awaji, Niigata Chuetsu, Noto, Niigata Chuetsu Offshore, and/or Great East Japan Earthquakes). The definition of “damaged” specifically referred to the authority’s need to provide nutrition assistance to the affected populations. If the authority had been damaged in any of the earthquakes, it was considered to have had an “experience of being affected by disaster.”

The second section had four questions focused on the gathering and distribution of disaster information, as follows:

Q1. Please choose the communication tools that are shown in your local disaster management plans, guidelines, or manuals (hereafter, local disaster management plans) as the means of communication with counterparts in emergencies. (The eight choices offered for this question are shown in Table 1.)

Q2. Do you have systems to collect information about damage to roads and railroads, traffic issues, the number of affected residents, damage to electricity, gas, water supply, and sewage systems, damage to food stores, and the number of evacuees and with or without cooking equipment at each shelter?

Q3. Do you have information collection systems so that the specified food supply facilities can report on their damage promptly, and are these reporting forms given during normal times?

Q4. Please choose the communication tools used to inform residents about the disaster situation, mass food supply centers, etc. (The 12 choices given are shown in Table 5).

#### 2.2.2. Comparison between 2005 and 2013

In the 2005 survey, the questionnaire was administered only to prefectures and cities with health centers. Therefore, to compare the 2005 and 2013 survey results, only the authorities that had answered both surveys were analyzed. Only the 2013 data were used for all other analyses.

### 2.3. Statistical Analysis

Fisher’s exact test was used to examine the relationships between disaster experience (or not) and every other question, and the McNemar test was used for comparison between the 2005 and 2013 survey responses. All statistical analyses were conducted with SPSS 20 (IBM Japan, Tokyo, Japan), with the significance set at 5%.

### 2.4. Ethical Considerations

The study procedures in the 2005 and 2013 surveys were approved by the Ethics Committee of the National Institute of Public Health and Ochanomizu University (2013-32), respectively.

## 3. Results

Reponses for the 2013 survey were received from 42 prefectures, 59 cities with health centers, 18 specified districts in Tokyo Metropolis, and 1153 municipalities (71.2% of all authorities). Thirty-six prefectures and 41 cities with health centers, and 16 specified districts in Tokyo Metropolis answered the surveys in both 2005 and in 2013, therefore the answers were comparable, as 66.4% of authorities answered the 2013 survey and 81.6% answered the 2005 survey (municipalities were not included in the 2005 survey).

Table 1 shows the number and percentage of authorities that listed the counterpart disaster communication tools from their local disaster management plans. As can be seen, 86.7% responded that disaster management radio communications was the most prevalent emergency communication tool in their local disaster management plans; however only 6.2% mentioned using SNS, with the percentage being even lower in municipalities.

Table 2 shows the changes in communication tools listed in local disaster management plans before and after the Great East Japan Earthquake. As can be seen, there was a significant increase in the use of computer networks and cell phones (email) as emergency communication tools in local disaster management plans from 2005 to 2013.

The number of authorities that experienced a disaster between the 2005 and 2013 surveys was also determined. However, Fisher’s exact test showed no significant relationship between disaster experiences in that period and the communication tools listed in Table 2. A test was run to see whether there was any relationship between the authorities that answered both questionnaires in 2005 and 2013 and their disaster experience, but no significant relationship was found.

Table 3 shows the number and percentage of authorities that reported having systems to collect information on damage to roads and railroads, traffic issues, the number of affected residents, damage to electricity, gas, water supply, and sewage systems, damage to food stores, and number of evacuees and with or without cooking equipment at each shelter. It was found that the percentage of municipalities with systems to collect this type of information was significantly higher in those that had experienced disasters than those that had not. However, no statistical differences were found between prefectures, cities with health centers, and specified districts in Tokyo Metropolis with or without disaster experience.

Table 4 shows the number and percentage of authorities that had established information collection systems in normal times that required designated food supply facilities to report damage promptly. Significant differences were found only between prefectures that had and did not have disaster experience.

Table 5 shows the number and percentage of authorities that considered the use of the listed tools to distribute information such as damage to resident food supply facilities. Municipalities that did not have disaster experience chose significantly more resident communication devices as PR tools such as websites, publicity cars, and local media such as FM radio and TV than those without disaster experience, which tended to more often select publication paper.

## 4. Discussion

### 4.1. Emergency Communication Tools Listed in Local Disaster Prevention Plans

Disaster management radio communication was the most widely cited tool in local disaster management plans in both the 2005 and 2013 surveys (Table 2). As such communication was used as part of the 1964 Niigata Earthquake and 1968 Tokachi-oki Earthquake, administrative agencies such as the Ministry of Internal Affairs and Communications have been encouraging authorities to equip themselves with this option [13]. However, three inland cities in Akita Prefecture have announced plans to dismantle their disaster management radio communication by the end of 2020 due to the renewal costs, and plan to use community FM radio and SNS as substitutes [14]. The number of authorities using disaster management radio communication might decrease in the future since community FM radio and SNS are used in normal times and are familiar and easy to use and maintain.

Although digital disaster management radio communication enables communication in both directions at the same time, only 22.9% of portable systems have been digitized [6]. Renewal of traditional communication tools has been shown to be vital in dealing with recent disasters. As with the food stockpiles, which need to be replaced every three to five years [15], communication tools also require periodic maintenance to ensure that they work properly, and it is also vital to conduct emergency training to ensure that the relevant people are able to use these communication devices [16].

Only 8% of authorities had SNS communication included in their local disaster management plans in the 2013 survey (Table 1). The most important SNS characteristic is that it allows everyone to be information senders, which means that the authorities can receive information from areas that are hard to reach. The Tokyo Metropolitan Government noted that SNS was a useful information-gathering and -sending tool in its “Disaster Readiness Guide” booklet [17]. Osaka City was the first to use SNS (LINE) to share information during its emergency training [18]. However, while this new information technology could be very useful in emergencies, the public needs to be educated on its use to ensure that it is only used to transmit true information and not rumors. US government agencies have also been using social media platforms for disaster communication, and have established social media networks in preparation for disasters, and guidelines on social media use during disasters [19]. We suggest that the Japanese government also issue official guidelines for social media use during disasters in the future.

### 4.2. Systems to Collect Information from Shelters and Communities During Disasters

In the Great East Japan Earthquake, there was a mismatch between the needs of those who were affected and the assistance that was provided. As a result, the Disaster Countermeasures Basic Act was amended, which included a reexamination of the way information is collected [20]. Although municipalities are the front-line disaster response organizations, only 25.5% reported having systems to collect information from shelters and communities (Table 3). While push operations for emergency logistics were used in Kumamoto Earthquake, the lack of information caused confusion in the affected authorities, the central government, and the Kumamoto Prefecture [21]. Fundamental information such as the number of evacuees at each shelter is essential in order to provide appropriate assistance. Although the Kumamoto Earthquake occurred three years after the amendment of the Disaster Countermeasures Basic Act, the confusion showed that the way of collecting information had much room for improvement.

Although the municipalities were not asked why they did not have systems to collect information from shelters and communities even though they are responsible for providing direct assistance to residents, cost could be the key factor, as it is with the keeping and maintenance of food stockpiles [12]. However, compared to disaster management radio communication or satellite telephone, SNS would be a cheaper option. Many in the Internet generation born between 1980 and 1990 are now parents and know how to receive and send information via SNS [22]. For example, photos could be taken of the food being served in shelters and sent to the municipalities, and then this information could be used to conduct triage on the shelters and determine which ones should be assisted first. SNS has already been used by civilians in disaster situations; for example, compared to the 2017 rain disaster, twice as many rescue requests were sent with the #Rescue Twitter hashtag in the 2018 West Japan heavy rain disaster [23], which proved that this information source could be valuable. We suggest, however, that people refrain from unnecessary posting in emergencies since real rescue requests could be buried in so much posted information.

### 4.3. Information Collection Systems Should Require Food Supply Facilities to Report Damage

All public health centers require that specified food supply facilities submit nutrition management reports every few months [24], which includes reporting on their preparedness for disasters, such as ensuring adequate food stockpiles and having a disaster response manual. Administrative dietitians working for public health centers check the reports and provide technical advice. If they distribute damage report forms during normal times, these could be included in facility manuals and be submitted promptly when a disaster occurs. However, only 36.4% of the prefectures with disaster experience had a food supply facility information collection system.

In the Great East Japan Earthquake, the food supply facilities in Miyagi Prefecture had to report on the meals that were offered [25]. However, as the dietitians working in the facilities had to record each menu by hand, it was time-consuming. As a result, the Miyagi prefectural government developed a checklist for the specified food supply facilities, which included crisis questionnaire items such as management structure, methods for collecting information, and hygiene management.

### 4.4. Tools to Distribute Information to Affected Residents

As outlined in the Disaster Counter Basic Act, when disasters occur, authorities need to collect and convey information to residents, which is important for two reasons [26]. First, the information can save lives; for example, evacuation information conveyed through broadcast disaster management radio communication helped disaster victims quickly escape in Onagawa Town, Miyagi Prefecture [6]. Second, information can bring disaster victims a feeling of relief especially if there is little specific information on the local area damage and it is difficult to offer emotional support [27]. However, in the Great East Japan Earthquake, information sharing was insufficient. The Ministry of Internal Affairs and Communications reported that 53.8% of victims and volunteers claimed that they were unable to get enough information about the civil service operations [11].

Most prefectures (82.9%) reported that they used websites to distribute information to affected residents (Table 5). Prefectures can more easily update website information than other authorities as they have more employees and would not be as confused as affected municipalities, and as they are responsible for logistical support in affected municipalities and need to directly communicate with central government ministries and agencies, they are able to more easily access external supporting information easily and put the new information on the website.

Municipalities, however, tended to use publicity cars to distribute information to affected residents as they are the closest authorities to the residents. Unless the roads are destroyed, publicity cars can deliver information to people who have lost power or have damaged communication devices. In the Niigata Chuetsu Earthquake, even though the city posted information on its website, households with elderly residents were unable to access it because of their computer illiteracy; therefore, in disasters, the old-fashioned ways of communicating are often more useful. For example, Yokohama City reported that information publications were useful in providing information to older people [28]. We suggest having many options for dispersion of risk as well as information distribution tailored to the characteristics of residents.

### 4.5. Limitation

This is a complete survey and we got high response rates (88.2% in 2005 and 71.2% in 2013) compared to other complete postal surveys [29,30]. For the comparison between the surveys, however, we analyzed only authorities that answered both surveys resulting in reduced sample size and selection bias.

## 5. Conclusions

The following conclusions were made from the disaster communication survey results. The most common disaster communication device at all authority levels in both the 2005 and 2013 surveys was disaster management radio communications. There were significantly more shelter information collection systems in municipalities that had been affected by disasters. Even though it is vital that food supply facilities report regularly to public health centers, only 6.7% of cities with public health centers and 12.2% of prefectures reported having food supply facility damage report systems. While websites were widely used by prefectures to convey new information, publicity cars were more widely used in municipalities.

## Figures and Tables

**Table 1:** Emergency communication tools listed in local disaster management plans, guidelines, or manuals. Data from 2013 survey.

Means of Communications	Prefectures (n = 41)		Cities with Health Centers and Special Wards (n = 73)		Municipalities (n = 1096)		Total (n = 1210)	
	n	%	n	%	n	%	n	%
Public telephone	5	12.2	16	21.9	190	17.3	211	17.4
Emergency telephone	17	41.5	40	54.8	553	50.5	610	50.4
Computer network	27	65.9	43	58.9	469	42.8	539	44.5
Cell phone (email)	24	58.5	45	61.6	565	51.6	634	52.4
Disaster management radio communication	39	95.1	68	93.2	942	85.9	1049	86.7
Satellite telephone	35	85.4	45	61.6	539	49.2	619	51.2
Social networking service (Twitter, Facebook, LINE)	10	24.4	19	26.0	68	6.2	97	8.0
Other	5	12.2	20	27.4	137	12.5	162	13.4

**Table 2:** Changes in means of communications shown in local disaster management plans, guidelines, or manuals before and after the Great East Japan Earthquake.

Means of Communication	2005		2013		P
	n	%	n	%	
Public telephone	19	21.3	14	15.7	0.42
Emergency telephone	48	53.9	43	48.3	0.56
Computer network	37	41.6	54	60.7	0.02
Cell phone (email)	36	40.4	56	62.9	<0.01
Disaster management radio communication	79	88.8	82	92.1	0.63

In total, 36 prefectures, 41 cities with health centers, and 16 specified districts in Tokyo Metropolis that answered both questionnaires in 2005 and 2013 were analyzed but four authorities did not answer this question; changes between 2005 and 2013 were examined using McNemar test.

**Table 3:** Authorities with systems to collect information from shelters and communities during disasters. ^1^ Data from 2013 survey.

Do You Have Systems to Collect Information from Shelters and Communities During Disasters?	Prefectures (n = 41)							Cities with Health Centers and Special Wards (n = 71)							Municipalities (n = 1117)						
	Experience of being Affected by Disasters							Experience of being Affected by Disasters							Experience of being Affected by Disasters						
	Have		None		Total		P	Have		None		Total		P	Have		None		Total		P
	n	%	n	%	n	%		n	%	n	%	n	%		n	%	n	%	n	%	
Yes	8	72.7	11	36.7	19	46.3	0.08	6	85.7	32	50.0	38	53.5	0.11	36	39.6	249	24.3	285	25.5	<0.01
No	3	27.3	19	63.3	22	53.7		1	14.3	32	50.0	33	46.5		55	60.4	777	75.7	832	74.5	
Total	11	100	30	100	41	100		7	100	64	100	71	100		91	100	1026	100	1117	100	

The number of authorities with information collection systems was compared between those with and without disaster experience using Fisher’s exact test; ^1^ Information collected included damage to roads and railroads, traffic issues, number of affected residents, damage to electricity, gas, water supply, and sewage systems, damage to food stores, and the number of evacuees and with or without cooking equipment at each shelter.

**Table 4:** Authorities with information collection systems that had “specified food supply facilities” reporting damage promptly by distributing reporting forms during normal times. Data from 2013 survey.

Do You Have Information Collection Systems that Had “Specified Food Supply Facilities” Reporting Damage Promptly by Distributing Reporting Forms during Normal Times?	Prefectures (n = 41)							Cities with Health Centers and Special Wards (n = 75)							Municipalities (n = 1113)						
	Experience of being Affected BY Disasters							Experience of being Affected by Disasters							Experience of being Affected by Disasters						
	Have		None		Total		P	Have		None		Total		P	Have		None		Total		P
	n	%	n	%	n	%		n	%	n	%	n	%		n	%	n	%	n	%	
Yes	4	36.4	1	3.3	5	12.2	0.01	1	14.3	4	5.9	5	6.7	0.40	6	6.6	59	5.8	65	5.8	0.65
No	7	63.6	29	96.7	36	87.8		6	85.7	64	94.1	70	93.3		85	93.4	963	94.2	1048	94.2	
Total	11	100	30	100	41	100		7	100	68	100	75	100		91	100	1022	100	1113	100	

Number of authorities with information collection systems was compared between those with and without disaster experience using Fisher’s exact test.

**Table 5:** Authorities that considered the use of listed tools to distribute information such as disaster damage by food provision to residents.

Means of Communication	Prefectures (n = 41)		Cities with Health Centers (n = 73)		Municipalities (n = 1119)	
	n	%	n	%	n	%
Website	34	82.9	56	78.9	^a^775	69.3
Publication car	18	43.9	56	78.9	^a^947	84.6
News board	11	26.8	32	45.1	352	31.5
Publication paper	15	33.6	33	46.5	^b^445	39.8
Acknowledgment by neighborhood association	14	34.1	36	50.7	675	60.3
Disaster management radio communication	17	41.5	57	80.3	882	78.8
TV, radio, newspaper	32	78.0	58	81.7	554	49.5
Request for broadcasting organization	24	58.5	42	59.2	403	36.0
Local media (FM radio, CATV)	20	48.8	49	69.0	^a^475	42.4
Social networking service (Twitter, Facebook, LINE)	16	39.0	33	46.5	220	19.7
Others	4	9.8	8	11.3	118	10.5
Did not consider	4	9.8	2	2.8	35	3.1

^a^ Larger percentage of municipalities without experience of being affected by disasters chose the item than those without experience (*p* < 0.05); ^b^ Larger percentage of municipalities with experience of being affected by disasters chose the item than those with experience (*p* < 0.05).
